# Internet-based survey evaluating the impact of ground substrate on injury and performance in canine agility athletes

**DOI:** 10.3389/fvets.2022.1025331

**Published:** 2022-10-17

**Authors:** Isabel A. Jimenez, Sherman O. Canapp, Monica L. Percival

**Affiliations:** ^1^Veterinary Orthopedic and Sports Medicine Group, Annapolis Junction, MD, United States; ^2^Department of Molecular and Comparative Pathobiology, The Johns Hopkins University School of Medicine, Baltimore, MD, United States; ^3^Canapp Sports Medicine LLC, Oakland, MD, United States; ^4^Clean Run Magazine, South Hadley, MA, United States

**Keywords:** canine agility, agility, medial shoulder syndrome, orthopedics, sports medicine, substrates, injury

## Abstract

Agility is a rapidly growing canine performance sport worldwide, yet the literature is sparse regarding the impact of ground substrate on performance and injury. Approximately 1/3 of dogs participating in agility trials will experience a performance-related injury. The impact of ground material has been well-documented in racing greyhounds, equine athletes, and humans, but has been minimally investigated in agility dogs. In this retrospective, cross-sectional study, 300 respondents (handlers, owners, and trainers) of 308 agility dogs completed an online survey regarding their dog's training and competition regimen, history of injury, perceived association between injury and substrate and/or agility obstacle, markers of decreased performance (MDPs) observed on different substrates, and changes to routine following injury. 35.7% of dogs sustained a training injury (TI) and 11.2% sustained a competition injury (CI). The most commonly reported sites of injury were the shoulder (TI: 33.9%, CI: 25.4%), forelimb digits (TI: 14.7%, CI: 11.9%) and iliopsoas muscle (TI: 11.9%, CI: 13.6%). Dogs most commonly trained on natural grass (85.3%), artificial turf (50.8%), and dirt (34.5%). Significantly fewer MDPs were observed on natural grass than any other substrate except dirt. Significantly more MDPs were noted on rubber mat compared to natural grass, artificial turf, dirt, sand, or foam mat. Rubber mat had the highest Incidence Proportion (IP) (32.0%) of TI and was perceived to be related to TI in 87.5% of cases. Obstacles perceived to be associated with injuries included jumps (TI: 37.5%, CI: 27.8%), contacts (TI: 29.7%, CI: 22.2%), weaves (TI: 11.9%, CI: 13.9%), and tunnels (CI: 25.0%). Overall, agility dogs were perceived to perform best on natural grass and dirt, while rubber mat was associated with injury and decreased performance. Respondents were willing to make significant alterations to their dog's routine due to a perceived association between substrate, injury, and performance. Further prospective studies are needed to assess the impact of substrate composition and maintenance, and inform evidence-based recommendations to maximize performance and minimize performance-related injury in agility dogs.

## Introduction

Agility is one of the most popular and fastest-growing canine performance sports worldwide. Approximately 30–40% of dogs participating in agility trials will experience a performance-related injury (1–4). Severe injury has been reported in 15.0% of agility dogs (3). In one study, following an orthopedic injury, 67.4% of agility dogs were reported to return to competition but 47% decreased in jump class (5). Another study reported that 10% of competition-related injuries resulted in retirement (4).

Over time, repetitive motions associated with athletic participation result in wear and tear on the joint of use, as has been demonstrated in human athletes (6, 7), horses (8, 9), and racing dogs (10). Excessive force on a joint can result in acute injury or contribute to development of chronic injuries, such as tendinopathies and osteoarthritis, with secondary impacts on other joints over time due to compensatory loading.

In canine agility athletes, the forelimb, and specifically the shoulder, is the most common site of injury (1, 2, 4, 11). The forelimbs carry 60% of a dog's body weight during the stance phase, and are subject to high peak vertical forces when landing from a jump (12). In addition, when landing from a jumping turn, the forelimbs experience higher lateral forces compared to the hindlimbs (13). Repetitive extension during jumping or sprinting is thought to contribute to supraspinatus tendinopathy, and repeated or excessive shoulder abduction in dogs making rapid sharp turns can result in injury to the medial compartment. The most common injuries sustained during canine agility are muscular and tendon injuries (1, 2, 14). Injuries to the digits and iliopsoas muscle are also common in agility dogs (11, 15, 16). Previously reported risk factors for performance-related injury in agility dogs include the dog's level of experience, type of obstacle, prior injury, Border Collie breed, and handler experience (1, 2, 17–20).

The impact of ground substrate on performance and risk of injury has been well-documented in human athletes (21, 22) and horses (23–25), and has also been investigated in racing greyhounds (26, 27). However, the literature is sparse with regards to the impact of ground substrate on performance and injury in canine agility athletes. Several recent survey-based studies have evaluated ground material as a risk factor for training and/or competition injuries in agility dogs (3, 4, 16, 28), but to the authors' knowledge, no studies have evaluated the relationship between ground material and performance deficits during agility competition.

**Figure 1:**
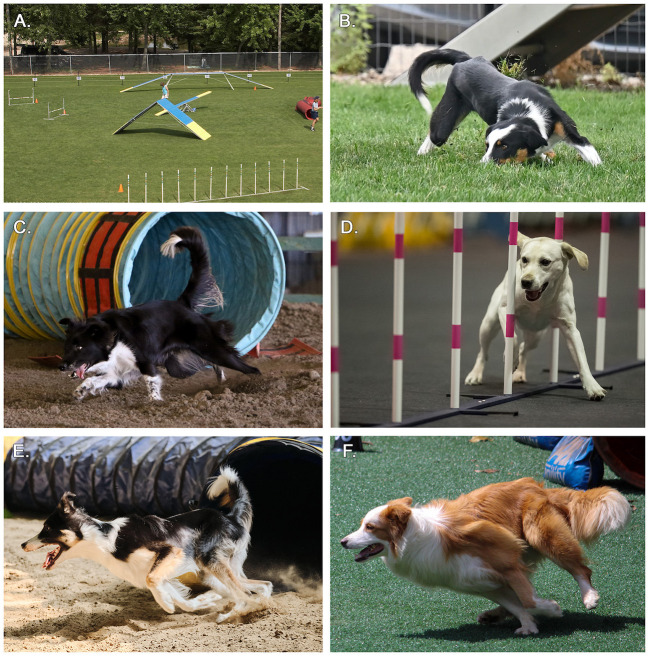
Examples of canine agility training and competition venue, obstacles, and substrates. **(A)** An outdoor competition venue with natural grass substrate, showing common obstacles (including teeter, dog walk, weave poles, A-frame, jump, and tunnel). A-frame, dog walk, and bar jumps have been associated with a higher rate of performance-related injuries. **(B)** A dog slips on natural grass on an outdoor agility course. Natural grass is one of the most common substrates used in canine agility. Weather, temperature, time of day, and drainage can affect performance on outdoor substrates. **(C)** A dog exits a tunnel obstacle and slips in deep, dry, uneven dirt. Dirt is a common substrate used in both indoor and outdoor agility courses. Depth, texture, and moisture should be consistent throughout the substrate to provide appropriate impact resistance while preventing excessive shifting of the substrate. **(D)** A dog executes weave poles on rubber mat and exhibits shoulder abduction. Shoulder abduction can contribute to the development of medial shoulder syndrome. The forelimb, and specifically the shoulder, is the most common site of injury in canine agility athletes. In the current study, rubber mat was associated with a higher incidence proportion of training injuries and decreased performance compared to other substrates. **(E)** A dog exits a tunnel at high speed. The sand substrate is uneven and the dog's inside hind limb sinks into the substrate. The continued use of substrate throughout the day can affect terrain composition and maintenance, potentially predisposing dogs to injuries during runs occurring later in the session. **(F)** A dog takes a running turn on artificial turf, demonstrating hyperextension of the digits of the inside forelimb. Forelimb digit injuries are common in agility dogs. Artificial turf may require replenishment of infill over time, and filling types appear to influence the rate of injury in prior literature.

Common substrates used during canine agility training and competition include natural grass, artificial turf, dirt, and sand, as well as various types of foam and rubber mat (Figure 1). Slips and falls during agility events occur quickly and are difficult to capture in still photographs, but can be appreciated on videos (Supplemental Video 1). Substrate composition and quality may influence the risk of slips, falls, or other injuries associated with agility. Stiffer (less compliant) flooring absorbs less energy, thus transferring more force back into the dog's musculoskeletal structures (10, 29). Conversely, a high-quality floor provides good traction for turns and jump-offs and acts as a shock absorber for landing after a jump. The optimal flooring would therefore minimize injury while maximizing performance.

In the authors' experience, the clinical presentation of musculoskeletal injuries in canine athletes often differs from that of the general pet population in that many work-related injuries in agility dogs are more often associated with decreased performance rather than overt lameness. Handlers may identify subtle performance deficits during competition that are not appreciated on visual gait evaluation outside of competition or on physical examination. For example, dogs with shoulder injuries may knock bars, take wide sweeping turns, or pull out of weaves, due to decreased shoulder extension and shortened step length. In this study, we utilized an internet-based survey of trainers, handlers, and owners of agility dogs with the goal of characterizing relationships between training surfaces and performance-related injury or markers of decreased performance (MDPs) in canine agility athletes. We also hypothesized that training and competing on a variety of surfaces would be protective against injury, as these dogs' joints would undergo diverse loading forces.

## Materials and methods

### Participants

Participants (handlers, trainers, and owners of canine agility athletes) were recruited through social media (Facebook, Facebook Inc, Menlo Park, CA), electronic email lists and newsletters, and an online canine agility magazine (Clean Run Magazine, South Hadley, MA). A brief statement accompanied the link to the survey to identify the purpose of the study and recruit respondents. Survey responses were accepted between the dates of September 7, 2020 to November 8, 2020.

### Survey

A 26-item questionnaire was developed through a free online survey platform (SurveyMonkey, Momentive, San Mateo, CA) utilizing input from professionals in canine surgery, sports medicine and rehabilitation, and agility training. Each survey response corresponded to one individual dog. Survey questions included free-text, simple categorical, and multiple categorical responses. Surveys were available in English. The survey questions are available in the Supplementary material.

Respondents provided contact information, their state and country of residence, their dog's signalment (breed, sex, reproductive status, and approximate age in years), and information about their dog's training regimen (including months/year, days/week, and hours/day spent training on agility obstacles, and percentage of time spent training indoors) and competition regimen (including years in competition, trials per year entered, agility level, agility classes, and trials typically entered). Respondents identified the percentage of time the dog typically spends training on each of nine common substrates (natural grass, artificial turf, dirt, sand, foam mat, rubber mat, wood mulch, pea gravel, and poured rubber) and were given the option of “Other” in which to enter a free-text response. Respondents also entered a free-text response to identify concurrent canine sports in which their dog participates. Due to the SARS-CoV-2 pandemic resulting in changes in training and competition, clients were instructed to answer questions for a “typical year.”

Separate sections were used to evaluate injuries sustained during training and injuries sustained during competition. Respondents were asked if their dog had ever sustained an injury (yes or no). If multiple injuries had been sustained, respondents were asked to choose one injury on which to focus for the survey. Respondents provided information about their dog's official diagnosis, if any; body site of injury; substrate in use at the time of injury; and the respondent's perception of whether the injury was related to a substrate and/or obstacle.

Incidence Proportion (IP) was calculated to account for the expected increased number of injuries occurring on substrates that are more commonly used. IP was defined as the number of dogs with training injuries (TI) sustained on a given substrate, divided by the number of substrate-exposures. The number of substrate-exposures was defined as the number of dogs reported to train on a given substrate.

Respondents ranked the nine core substrates from 1 to 10, with 1 indicating the surface on which their dog performs best. Substrates that did not apply to their dog were left out. For each substrate, respondents also selected any MDPs they had observed on that substrate. For each individual dog, performance was only evaluated for substrates that the dog currently or previously trained on. Individual MDP score was calculated for each dog (Supplementary Table 1). Mean substrate MDPs were calculated for each substrate.

Respondents provided a free-text response describing how, if applicable, their dog's training or competition regimen have changed since an injury was sustained, and how their decision to train on certain substrates has been influenced by their dog's performance.

Respondents' identities were anonymized prior to data analysis but contact information was retained in a confidential file for follow-up communication. The estimated time for survey completion was 23 min. If questions were omitted or filled out incorrectly (e.g., percentages that summed to over 100%) by the respondent, an attempt was made to contact the respondent by email to correct the answer. 40 respondents were contacted by email for additional information. Respondents were given at least 30 days to respond; if no response was received, incomplete or incorrect data was omitted from the relevant sections prior to analyses.

### Statistical analysis

Commercial software was used for statistical analysis (Graphpad Prism 9.1.0; GraphPad Software, San Diego, CA). Unpaired two-tailed *t*-tests were used to assess the effect of quantitative continuous variables on the incidence of TI and CI, at a 95% confidence interval. To analyze the effects of different substrates on MDPs between individuals, we implemented a mixed effects model using a compound symmetry covariance matrix, fit using Restricted Maximum Likelihood (REML) and implementing the Geisser-Greenhouse correction. Tukey's multiple comparisons follow-up test was performed for pairwise comparison of mean MDP for each substrate and multiplicity adjusted *P*-values were calculated. For binary variables, 2 x 2 contingency tables were evaluated using a Fisher's Exact Test. Relative Risk (RR) was defined as the proportion of dogs with a history of injury that were positive for the parameter of interest, relative to the proportion of dogs with a history of injury that were negative for the parameter of interest. Content analysis of free-text responses was performed to standardize responses, produce qualitative categories and identify common themes.

## Results

### Demographics

A total of 300 respondents participated in the survey and provided data on 308 dogs. Most (72.0%) respondents lived in the United States (41 states), followed by Canada (18.7%) and the United Kingdom (4.3%). 43.8% of dogs in the study were female and 56.2% were male, and ages ranged from 1.25 to 16.5 years (median: 6.58 years).

Sixty two dog breeds were represented, with the most common breeds being the Border Collie (31.5%), mixed breed dogs (10.4%), Shetland Sheepdog (7.1%), Australian Shepherd (6.8%), Golden Retriever (5.2%), and Labrador Retriever (3.6%). All AKC breed groups were represented, with most dogs in the Herding group (56.5%) followed by the Sporting group (15.6%).

Most dogs (57.8%) concurrently participated in a canine sport or activity other than agility. The most common concurrent sports were rally (36.0%) and obedience (34.3%), followed by scentwork/nosework (27.0%), dock diving (22.5%), and barn hunt (14.6%). There was no significant effect of participation in concurrent sports on the incidence of TI (*p* = 0.0616) or CI (*p* = 0.4744) when the number of concurrent sports was evaluated as a quantitative variable, nor was there an impact of concurrent sport participation on Relative Risk (RR) of injury (Table 1).

**Table 1:** Relative risk for training injury and competition injury in canine agility athletes.

**Training injuries**		
**Parameter**	**RR** ^*^	***p*** **-value** ^†^
Age (<6 years old vs. ≥6 years old)	1.2558	0.1809
Sex (female vs. male)	0.9611	0.8111
Concurrent sports (No vs. Yes)	0.7989	0.1491
Substrate diversity (≤3 substrates vs. ≥4 substrates)	1.0593	0.8465
**Competition injuries**		
**Parameter**	**RR** ^*^	***p*** **-value** ^†^
History of training injury (No vs. Yes)	2.2846	0.0005
Age (<6 years old vs. ≥6 years old)	5.7935	<0.00001
Sex (female vs. male)	0.9904	1.0000
Concurrent sports (No vs. Yes)	1.0267	1.0000
Substrate diversity (≤3 substrates vs. ≥4 substrates)	1.1795	0.6362

* Relative Risk (RR) was defined as the proportion of dogs with a history of injury that were positive for the parameter of interest, relative to the proportion of dogs with a history of injury that were negative for the parameter of interest. RR was calculated from 2 × 2 contingency tables.

† The significance level was α = 0.05. The p-value was calculated using the Fisher's Exact Test.

### Training regimen and training injuries

The majority (91.2%) of dogs were reported to train, or to have trained prior to retirement, in North America, followed by Europe (7.2%), Africa (1.3%), and Australia (0.98%). The training regimen in a typical year was evaluated for 301 dogs; 7 dogs were retired and therefore excluded. Most dogs trained 10–12 months/year (85.4%) or 6–9 months/year (12.3%). Most dogs trained ≤ 3 days per week (81.4%). Most dogs also trained for ≤ 30 min per session (51.2%) or between 30 and 60 min per session (43.2%). On average, dogs spent a similar amount of time training indoors (48%) and outdoors (52%). 17.6% of dogs trained exclusively outdoors and 10.6% of dogs trained exclusively indoors.

**Figure 2:**
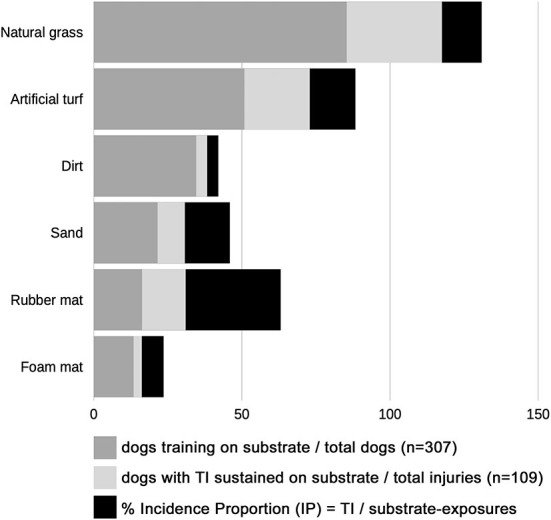
Training frequency and injuries sustained on most common substrates. The substrates most commonly included in the training regimen were natural grass (85.3%), artificial turf (50.8%), dirt (34.5%), sand (21.5%), rubber mat (16.3%), and foam mat (13.4%) (*n* = 307). The most common substrates in use when training injuries (TI) were sustained were natural grass (32.1%), artificial turf (22.0%), rubber mat (14.7%), and sand (9.2%) (*n* = 109). Incidence Proportion (IP) was calculated for each substrate. IP was defined as the proportion of number of dogs that sustained training injuries (TI) on the substrate, out of the number of substrate-exposures (number of dogs reported to train on that substrate). Rubber mat had the highest IP (32.0% [16/50]) while dirt had the lowest IP (3.77% [4/106]).

A total of 22 distinct substrates were reported as part of the training regimen of 307 dogs. In addition to the nine core substrates, additional substrate categories emerged, including sand mixtures, stone/gravel mixtures, dirt mixtures, and clay. On average, an individual dog trained on 2.3 different substrates. The substrates most commonly included in the training regimen were natural grass (85.3%) and artificial turf (50.8%), followed by dirt (34.5%), sand (21.5%), rubber mat (16.3%), and foam mat (13.4%) (Figure 2). Natural grass occupied the largest proportion of training time for dogs that included grass in their training regimen (51.8%), followed by artificial turf (50.9%), wood mulch (39.8%), foam mat (36.1%), rubber mat (33.0%), dirt (32.1%), and sand (24.8%) (Supplementary Table 1). Respondents whose percentage time response did not add up to 100% were contacted to correct their answers; 12 individuals did not respond to the request and their responses to this section were excluded.

**Figure 3:**
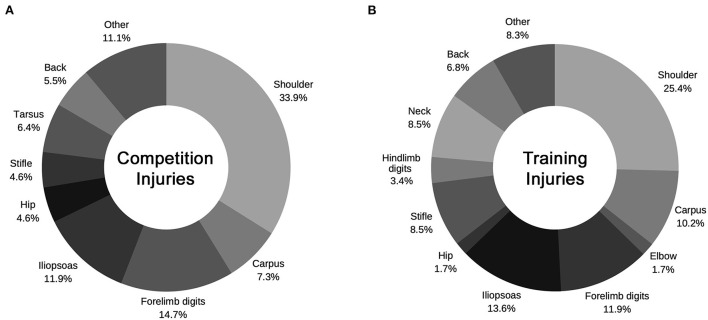
Most common body sites sustaining injury during training and competition in canine agility athletes. **(A)** The most common sites injured during training (*n* = 109) were the shoulder (33.9%), forelimb digits (14.7%), iliopsoas muscle (11.9%), carpus (7.3%), and tarsus (6.4%). **(B)** The most common sites injured during competition (*n* = 59) were the shoulder (25.4%), iliopsoas muscle (13.6%), forelimb digits (11.9%), carpus (10.2%), stifle (8.5%), neck (8.5%), and back (6.8%).

35.7% (110/308) of dogs sustained TI. Detailed information on 109 TIs was available. Most TIs (94.5%) involved the limbs; of these, the forelimbs were most often affected (64.2%). 6.8% of dogs sustained TI to more than one limb. The most common body sites injured during training were the shoulder (33.9%), forelimb digits (14.7%), iliopsoas muscle (11.9%), carpus (7.3%), and tarsus (6.4%) (Figure 3A).

There was no effect of training schedule on TI, including months training per year (*p* = 0.0849), days training per week (*p* = 0.421), hours training per session (0.7788), and percentage of time spent training indoors (*p* = 0.9898). There was no impact of sex on the RR of TI or competition injury (CI) (Table 1).

**Figure 4:**
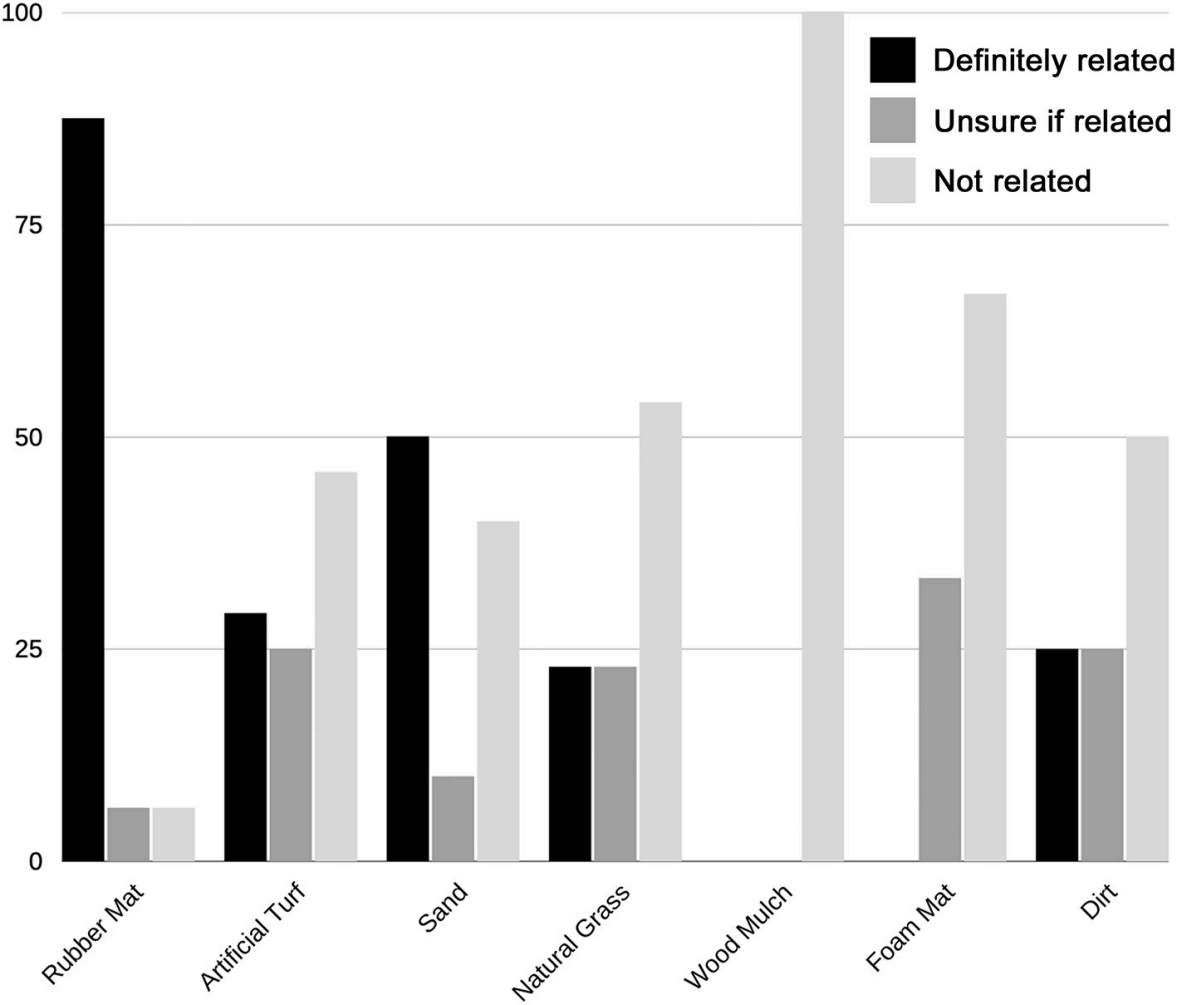
Percentage of training injuries (TI) perceived to be associated with each major substrate. Most TIs (*n* = 109) were perceived by respondents to be definitely (34.9%) or possibly (21.1%) related to the substrate. Of injuries sustained on the major substrates (rubber mat [*n* = 16], artificial turf [*n* = 24], sand [*n* = 10], natural grass [*n* = 35], wood mulch [*n* = 1], foam mat [*n* = 3], and dirt [*n* = 4]), rubber mat was most often perceived by respondents to be responsible for the injury.

Of the 109 TIs recorded, the most common substrates in use when TI was sustained were natural grass (32.1%), artificial turf (22.0%), rubber mat (14.7%), and sand (9.2%) (Figure 2). Rubber mat had the highest IP (32.0% [16/50]) while dirt had the lowest IP (3.77% [4/106]) (Supplementary Table 2). Most TIs were perceived by respondents to be definitely (34.9%) or possibly (21.1%) related to the substrate. Rubber mat was the substrate most often perceived by respondents to be responsible for the injury (87.5%) (Figure 4).

Over half (58.7%) of TIs were perceived to be associated with an obstacle. Of these, the obstacles most often associated with TIs were jumps (37.5%), contacts (29.7%) and weaves (11.9%). Contacts are agility obstacles with which the dog makes physical contact (running over or stopping on the obstacle), and in this study included A-frame, dog walk, teeter, and pause table (Supplementary Table 3).

### Competition regimen and competition injuries

Three hundred and three dogs were currently in competition or had previously been in competition. Five dogs had never competed and were excluded. Most (90.8%) dogs competed in North America and Europe (9.9%). Most dogs competed at the Masters/Senior (56.8%) and Champion (30.7%) levels, followed by Open/Advanced (15.2%), Excellent (13.5%), and Starters/Novice (13.2%). Most dogs competed in the Standard Agility (95.4%) and Jumping (94.1%) classes, followed by Games/Nonstandard classes (80.0%), Premier/International/Masters Challenge (44.6%), and Tournaments (35.3%). The most common agility trials attended were the AKC (62.4%), UKI (42.9%), and USDAA (35.0%), followed by the AAC (18.5%), NADAC (12.9%), and CPE (12.9%). Of the 196 dogs still in competition, in a typical year, 40.5% competed in 10–20 trials, 35.1% competed in <10 trials, 15.2% competed in 21–30 trials, 5.1% competed in 31–40 trials and 4.1% competed in >40 trials per year. CI were reported in 19.5% (59/303) of dogs currently or previously in competition. Most (84.8%) CIs involved the limbs; of limb injuries, the forelimbs were most often affected (62.7%). 5.1% of dogs sustained a CI affecting more than one limb. The most common body sites injured during competition were the shoulder (25.4%), iliopsoas muscle (13.6%), forelimb digits (11.9%), carpus (10.2%), stifle (8.5%), neck (8.5%), and back (6.8%) (Figure 3B).

Dogs with a history of CI participated in significantly more agility classes (3.64 ± 0.15) compared to dogs without a history of CI (3.29 ± 0.08) (*p* = 0.0432). There was no significant effect of the number of trials entered per year on the incidence of CI (*p* = 0.3933).

**Figure 5:**
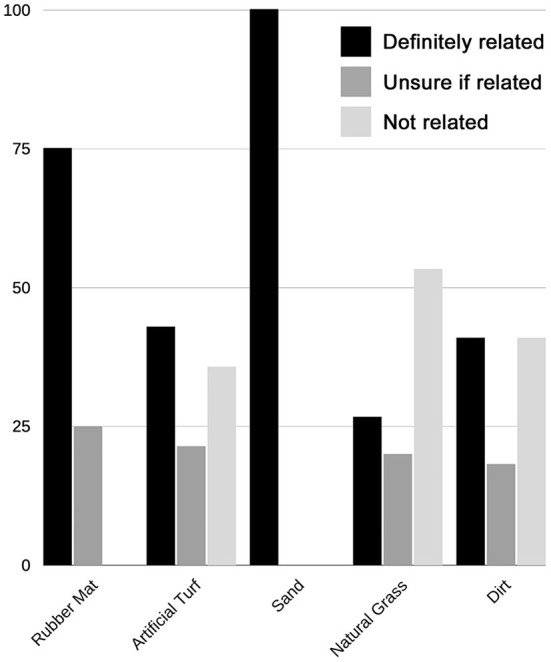
Percentage of competition injuries (CI) perceived to be associated with each major substrate. Most CIs (*n* = 59) were perceived by respondents to be definitely (40.7%) or possibly (20.3%) related to the substrate. Of injuries sustained on the major substrates (rubber mat [*n* = 4], artificial turf [*n* = 14], sand [*n* = 2], natural grass [*n* = 15], and dirt [*n* = 22]), sand (100%), rubber mat (75%), and artificial turf (42.9%) were most often perceived by respondents to be responsible for the injury.

The most common substrates in use when CI was sustained were dirt (37.3%), natural grass (25.4%), artificial turf (23.7%) and rubber mat (6.8%) (Supplementary Table 4). IP could not be calculated for CI, as the number of substrate-exposures is unknown. Most respondents perceived that CI was definitely (40.7%) or possibly (20.3%) related to the substrate. The substrates most commonly perceived by respondents to be responsible for CI were sand (100%) and rubber mat (75.0%) (Figure 5).

Most CIs (61.0%) were also perceived to be associated with an obstacle. Of these, the obstacles most often associated with CI were jumps (27.8%), tunnels (25.0%), contacts (22.2%), and weaves (13.9%) (Supplementary Table 5).

Approximately half (44.9%) of the dogs in this study sustained either TI or CI, and 11.2% of dogs sustained both TI and CI. Dogs with a history of TI were 2.28 times more likely to also have a history of CI compared to dogs without a history of TI (*p* = 0.0005) (Table 1).

When age was evaluated as a quantitative variable, older dogs were significantly more likely to have suffered a CI (*p* = 0.0001); the mean age for dogs with a history of CI was 8.63 ± 0.29 years, compared to 6.45 ± 0.17 years in dogs without a history of CI. When age was evaluated as a binary variable, dogs ≥6 years of age were 5.79 times more likely to have a history of CI (*p* < 0.00001) (Table 1). The mean age of dogs with a history of TI was 7.26 years, while the mean age for dogs without a history of TI was 6.62 years, but this difference was not quite significant (*p* = 0.0500); there was no impact of age on the RR of TI.

Of the 136 dogs with a history of TI or CI, most respondents (66.9%) reported implementing changes to their dog's routine as a result of the injury (Table 2). The most common changes were completely discontinuing (34.1%) or decreasing the use of (45.0%) a particular substrate. 35.2% of respondents reported that substrate conditions were used to inform a decision to adjust training or withdraw from an event. Other changes included reducing overall training and trialing time (13.2%), avoiding certain obstacles (14.3%), and changing the type of agility trials that their dog entered (12.1%). 25.3% of respondents instituted more regular warmups and cooldowns, and 23.1% added more conditioning and core strengthening fitness exercises. Following recovery from the injury, 11.0% continued maintenance formal rehabilitation and 14.3% of dogs underwent at-home rehabilitation. 5 dogs (5.5%) were retired as a direct result of their injury.

**Table 2:** Most common modifications to training and competition regimens following performance-related injury (*n* = 308).

**Modification**	**# of dogs**	**% of dogs**
**Rehabilitation**		
Maintenance formal rehabilitation *(including chiropractic, formal massage, physical therapy, underwater treadmill, acupuncture, laser)*	10	10.99%
At-home rehabilitation *(including home exercises, massage,icing, stretching)*	13	14.29%
**Substrate use**		
Discontinue activity on certain substrates	31	34.07%
Reduce amount of time on certain substrates	41	45.05%
Evaluate substrate conditions (including depth, slipperiness) and adjust training or withdraw	32	35.16%
**Training and Competition Regimen**		
More regular warmup and cooldown	23	25.27%
Add core-strengthening and conditioning	21	23.08%
Reduce overall training and trialing time	12	13.19%
Change the type of trials entered (including changes in competition level or jump height, or discontinuing certain classes)	11	12.09%
Avoid or reduce time on certain obstacles	13	14.29%
Retirement	5	5.49%

### Performance assessment

**Figure 6:**
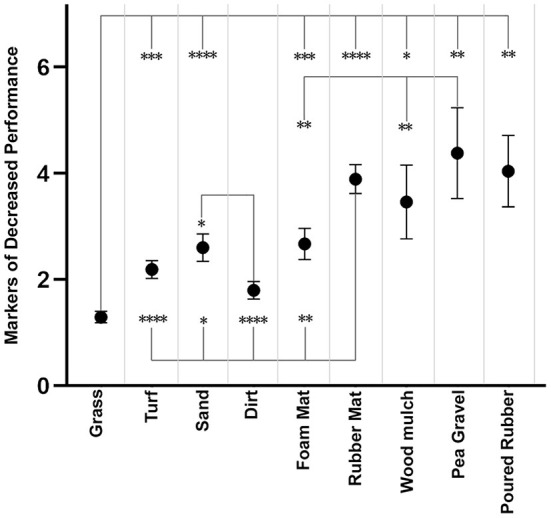
Mean markers of decrease performance and standard error for each substrate. Significant differences in markers of decreased performance (MDPs) between substrates during individual pairwise comparison are summarized with asterisks (*p-*value <0.05 = one asterisk, *p-*value <0.01 = two asterisks, *p-*value <0.001 = three asterisks, and *p-*value <0.0001 = four asterisks). Significantly fewer MDPs were observed on natural grass than any other substrate except dirt. Significantly more MDPs were noted on rubber mat compared to natural grass, artificial turf, dirt, sand, or foam mat. The significance level is α = 0.05.

When asked to rank their dog's performance on the core substrates on a scale from 1 to 10, on average, respondents subjectively perceived their dogs to perform best on natural grass (1.98), followed by artificial turf (2.24), dirt (2.47), sand (3.25), foam mat (3.57), rubber mat (4.18), wood mulch (4.89), poured rubber (6.70) and pea gravel (7.31). Respondents scored each substrate for MDPs noted while evaluating their dog's performance on that substrate. The substrate with the most performance evaluations was grass (278), followed by artificial turf (231), dirt (208), rubber mat (133), sand (126), and foam mat (89). Respondents recorded a lower MDP on natural grass (1.3) compared to any other substrate. Approximately half (50.4%) of dogs did not exhibit any performance deficits on natural grass (Table 3). Dirt had the second lowest mean substrate MDP (1.8). 42.3% (88/208) of dogs did not show any performance deficits on dirt. Respondents noticed significantly fewer MDPs on natural grass compared to rubber mat (*p* < 0.0001), sand (*p* < 0.0001), foam mat (*p* = 0.0003), artificial turf (*p* = 0.0005), poured rubber (*p* = 0.0019), pea gravel (*p* = 0.0073), and wood mulch (*p* = 0.0350) (Figure 6). Pea gravel (4.4), poured rubber (4.0), and rubber mat (3.9) had the highest MDPs. Rubber mat was associated with significantly more MDPs compared to natural grass (*p* < 0.0001), artificial turf (*p* < 0.0001), dirt (*p* < 0.0001), foam mat (*p* = 0.0020), and sand (*p* = 0.0320). Overall, there was a significant effect of substrate on mean MDP between individuals (*p* < 0.0001). Neither sex nor reproductive status had a significant effect on mean MDP between individuals.

**Table 3:** Markers of decreased performance (MDPs) by substrate.

**Substrate**	**All dogs (*n =* 308)**				**Dogs training on<3 substrates (*n =* 274)**				**Dogs training on≥ 4 substrates (*n = * 33)**				
	Total*	**No MDPs**		**Mean MDP**	**Total**	**No MDPs**		**Mean MDP**	**Total**	**No MDPs**		**Mean MDP**	***p-*value^†^**
		**n**	**%**			**n**	**%**			**n**	**%**		
Grass	278	140	50.4%	1.3	246	133	54.1%	1.2	31	6	19.4%	2.1	0.0121
Artificial turf	231	81	35.1%	2.2	201	77	38.3%	2.1	29	4	13.8%	2.6	0.3252
Sand	126	40	31.8%	2.6	111	39	35.1%	2.4	14	0	0%	4.3	0.0231
Dirt	208	88	42.3%	1.8	178	77	43.3%	1.7	30	11	36.7%	2.7	0.0236
Foam mat	89	26	29.2%	2.7	72	23	31.9%	2.7	17	3	17.7%	2.5	0.7398
Rubber mat	133	15	11.3%	3.9	111	10	9.0%	4.0	21	4	19.0%	3.8	0.8548
Wood mulch	26	7	26.9%	3.5	20	6	30.0%	3.3	6	1	16.7%	4.2	0.7634
Pea gravel	21	3	14.3%	4.4	16	3	18.8%	4.1	5	0	0%	5.4	0.5891
Poured rubber	23	1	4.4%	4.0	19	1	5.3%	4.0	4	0	0%	4.5	0.5192

^*^Refers to the total number of individual dogs for whom MDPs have been noticed for the given substrate.

^†^The significance level was α = 0.05. The p-value was calculated using an unpaired two-tailed T-test.

The average MDP score for dogs with a history of CI was significantly higher (2.80) than that of dogs without a history of CI (1.81) (*p* < 0.0001). Dogs with a history of TI also had a significantly higher average MDP score (2.39) compared to dogs without a history of TI (1.79) (*p* = 0.0011).

### Substrate diversity and modifications to substrate use based on performance

The impact of substrate diversity on injury was evaluated using two methods. When assessed as a quantitative variable (number of substrates reported in the dog's training regimen), there was no effect of substrate diversity on the incidence of TI (*p* = 0.1591) or CI (*p* = 0.5267). When substrate diversity was divided into binary categories—dogs with less substrate diversity (≤3 substrates in training regimen) vs. dogs with more substrate diversity (≥4 substrates in training regimen), there was no impact of substrate diversity on the Relative Risk of TI or CI (Table 1). The effect of substrate diversity on performance was evaluated as a binary categorical variable for each of the core ground materials (Table 3). Dogs with more substrate diversity (≥4 substrates in training regimen) had significantly higher mean MDP for natural grass (*p* = 0.0121), sand (*p* = 0.0231), and dirt (*p* = 0.0236).

Most respondents (58% [128/221]) indicated that their dog's performance influenced their decision to train on certain substrates. Two additional themes emerged from this free-text response; 7% (15/221) of respondents reported that they no longer train at certain facilities or enter certain trials due to prior experiences with the substrate at the venue, and 11% (24/221) of respondents reported that they perceive their geographic area lacks availability of different substrates to train or compete on, limiting their ability to make choices in substrate use.

## Discussion

Over 30% of agility dogs in this study had a history of TI, and nearly 20% had sustained a CI, consistent with rates of injury reported in prior literature (1, 2, 4, 30). Nearly half (44.9%) of all dogs in the study had sustained either TI or CI. This high rate of injury not only conveys morbidity for the individual patient but also carries consequences for the dog's ability to train and compete, resulting in both financial impacts and strain on the human-animal bond, when a high-drive animal is activity-restricted during recovery. Dogs with a history of TI were significantly more likely to have a concurrent history of CI, also consistent with the prior literature (18). Dogs with a history of injury also had significantly more performance deficits compared to dogs without a history of injury, which may reflect long-term decreased performance as a result of the injury. Alternatively, an external factor (such as less experience) may predispose dogs to both injury and poor performance, or respondents whose dogs had previously suffered injuries may have been more discriminating in assessing performance. While our results did not show an association between concurrent sports and risk of injury, a recent study reported a protective effect of participating in physically demanding sports on competition-related injury in agility dogs (4), suggesting an avenue for further research.

Forelimb injuries accounted for ~60% of the competition and training injuries in this study, with the shoulder comprising approximately 1/3 of TI and 1/4 of CI, consistent with prior literature (1, 2, 11).

Most TIs and CIs in our study were perceived to be obstacle-associated, with the most common obstacles being jumps, contacts, and weaves for both TIs and CIs, as well as tunnels for CIs. The predominance of jumps and contacts in obstacle-associated injuries is consistent with prior literature, in which A-frame, dog walk, and bar jumps are consistently associated with a higher rate of injury (1, 2, 4).

Jump height has been associated with increased injury risk (3). Obstacle height and the distance between obstacles has also been demonstrated to affect speed and landing angle (12). In addition, jump angle and landing angle may alter biomechanical forces. Söhnel et al. (20) reported variation in forelimb biomechanics between beginner and advanced agility dogs landing from a jump, resulting in higher limb compression and larger eccentric muscle contraction in beginner dogs. In a prior study, dogs with <4 years of agility experience had greater variability in head and neck position and exaggeration of the apex jump during runs over the A-frame (31), further suggesting that inexperience could contribute to obstacle-related injuries. Conversely, Söhnel et al. (13) reported that landing from a turn jump is a constrained motion with relatively little variation, regardless of individual techniques or skill level.

Agility dogs have different stepping patterns even when completing the same obstacles; individual variation in gait style may affect joint stress and biomechanics, potentially altering the risk of injury (32). In contact obstacles, the material from which the obstacle is constructed and the potential effect of weather conditions (i.e., if the obstacle is wet) may also impact the rate of injury associated with slipping on, or colliding with, an obstacle. In general, contact performance can require a dog to stop at the end of a contact (referred to as the “2 on 2 off position”), or perform a “running contact” in which the dog does not stop, and typically executes the contact at a much higher velocity. Beyond obstacle type, therefore, course design and order of obstacles can further influence the biomechanical forces on the limbs and back. Further research is needed to evaluate whether course design in combination with ground substrate and surface characteristics of contact obstacles can be used to reduce injury and improve performance in agility dogs. While tunnels have previously been infrequently associated with injury, Inkilä et al. (4) recently reported a higher rate of tunnel-associated injuries in a population of Finnish dogs, and suggested that increased tunnel speeds and more fixed tunnel attachments may have increased the hazards of tunnels over time.

More than half of respondents perceived injuries to be related to the substrate. The training regimens of dogs in this study included 22 substrates. The most common training substrates were natural grass (85.3%), artificial turf (50.8%), and dirt (34.5%), followed by sand, rubber mat, and foam mat. Because the use of different substrates was not uniform—many more dogs trained on natural grass than on wood mulch, for example—we expected that a higher overall number of TIs would occur on the more common substrates. To better illustrate for the reader the slips and falls occurring on different substrates, we requested that volunteers submit videos over social media. A subset of videos were complied and accompanied by freeze-frames of the slip or fall (Supplemental Video 1). Despite our results suggesting that rubber mat was associated with injury and decreased performance, none of the videos showed slips or falls on rubber mat. This further illustrates the fact that the number of falls observed (and captured on video) on a particular substrate will be skewed by the frequency of use of that substrate, and highlights the value of utilizing IP to normalize the rate of injury to the rate of substrate use. To account for this, IP was calculated to present the rate of injury on a substrate relative to the proportion of dogs training on that substrate. IP has not been utilized in other studies evaluating the proportion of injuries sustained on different surfaces in agility dogs (3, 4, 16), which may skew results toward more common surfaces.

In the current study, rubber mat had the highest IP (32.0%) for TI, more than twice that of any other substrate, indicating that a higher proportion of TIs occurred on rubber mat than on other surface (Figure 2). Rubber mat was also the substrate most often perceived to be responsible for TI and the second most often perceived to be responsible for CI, after sand. Based on our results, rubber mat was also associated with decreased performance compared to other substrates.

Pechette Markley et al. (3) recently evaluated the association between these same common surfaces and competition-related injury in agility dogs, reporting a lower risk of injury in dogs that had competed 6+ times on rubber mat, compared to dogs that had never competed on rubber mat or dogs that competed <6 times per year on rubber mat. It is possible that acclimation to rubber mat, through prior experience competing on that surface, conveys a protective effect. The need to acclimate before a protective effect is achieved could indicate that this material is less consistent in performance compared to other surfaces. Conversely, Inkilä et al. (4) evaluated dirt/sand and various types of artificial turf, and reported that 67.6% of injuries occurred on a surface on which the dog had trained or competed on at least weekly during the 3 months prior to injury, which suggests that injuries occurred despite the dog's familiarity with the surface.

Anecdotally, the use of rubber mat has declined in training and competition venues, as it is perceived to be slippery (3). We suspect that rubber mat exhibits variability in traction and consistency based on a variety of factors, including its age, maintenance, cleaning, and underlying padding, and therefore likely varies over time and between venues. In addition, factors such as the agility level or the order of obstacles on the course, and the consequent biomechanical demands on the athlete, could influence the relative impact of the substrate on injury risk. For example, a course that requires a dog to compete at higher speeds could be less forgiving if the traction provided by the substrate is not ideal.

It is difficult to assess the relationship between substrates and CI because the frequency of use of different competition substrates was not available in our study. For instance, while the substrates most often in use at the time a CI was sustained were dirt (37.3%), natural grass (25.4%), artificial turf (23.7%) and rubber mat (6.8%), it is possible that these are highly represented simply because these substrates are most commonly used in competition venues. There is a clear difference between the incidence of CI on different substrates and whether the substrates were perceived to be at fault. For example, approximately a quarter of CIs occurred on both natural grass and artificial turf. However, while only a quarter of CI sustained on natural grass were perceived to be secondary to the substrate, artificial turf was perceived to be responsible for 42.9% of CI on that substrate.

Respondents subjectively perceived their dogs to perform best on natural grass, followed by artificial turf and dirt. The fewest performance deficits were observed on natural grass and dirt, with approximately half of dogs exhibiting no performance deficits on these surfaces. A lower proportion of TIs were sustained on dirt compared to other surfaces. These results suggest that natural grass, dirt, and artificial turf may provide improved performance and reduced risk of injury. In a recent evaluation of common surfaces in agility competition, Fry et al. (16) reported that dogs who had ever competed on dirt or artificial turf were more likely to have reported an iliopsoas injury, compared to dogs with no history on that surface. However, that study did not evaluate the substrate in use at the time the injury was sustained, only the dogs' history of prior substrate use. Dirt and artificial turf are common substrates used in competition; dogs that have been to more competitions may experience a wider variety of substrates, but also may have more opportunities for injury. There is insufficient information provided regarding the model used to determine whether these potentially confounding effects were considered.

A major limitation of the current study is the inability to assess the preparation and maintenance of the ground material, which likely impacts the consistency and reliability of the flooring and could alter injury risk and performance quality. Racetrack surface composition and maintenance is a key element contributing to injury in racing greyhounds (26, 33–36). The kinematics of canine agility and jumping differ from those involved in racing, therefore the optimal ground material in greyhound racing does not necessarily directly translate to the optimal flooring to maximize performance and reduce injury in agility dogs. Additionally, life-threatening injuries are expected to be more common in racing greyhounds compared to agility dogs, therefore concerns over ground material are likely to be more focused on ameliorating fatality rather than preventing non-fatal injuries. Despite the clear differences between racing greyhounds and agility dogs, research on the impacts of track material in racing greyhounds offers potential insights into the importance of the track surface.

The racetrack is composed of a deep traction layer and an overlying absorptive layer. The depth of both layers must be consistent throughout the track. The absorptive layer must contain sufficient moisture to absorb the force of impact, but excessive moisture or inappropriate drainage can cause the superficial layer to shift or become uneven (27). If the superficial layer becomes too dry or compacted, the track becomes hard, resulting in higher impact resistance. Surfaces with less compliance transmit greater impact forces to the hind limb of racing greyhounds (10, 29). In racing greyhounds, particle size, moisture, and density of sand surfaces, as well as variability between different portions of the racetrack, could contribute to injury (37). It is highly likely that similar factors apply to canine agility. For example, the frequency of turnover throughout the day can change the quality of morning vs. afternoon runs. Inkilä et al. (4) reported that most TI occurred in the second half of the training session while most CI occurred during later runs, which could suggest that fatigue and/or changes in substrate condition throughout the day increase the risk of injury. Weather, temperature, time of day, and drainage can affect the moisture content and impact resistance of outdoor substrates, such as grass and dirt, while cleaning products can affect the texture of indoor surfaces, such as artificial turf, rubber mat, and foam mat. The age of the flooring, particularly artificial substrates that may require replenishment of infill or which may stretch or break down over time, may also affect substrate consistency and force transmission. Artificial turf filling types appear to influence the rate of injury; for example, in one study, 35.1% of injuries occurred on artificial turf with rubber filling compared to 12.6% of injuries on artificial turf with cork filling (4). Few manufacturers include specific maintenance recommendations as to the type or frequency of maintenance for different substrates, therefore maintenance is likely to vary significantly between, and among, training facilities and competition venues. In this study, the average dog trained on 2.3 substrates.

We had hypothesized that training on a diversity of substrates might be protective against injury, as these dogs would be more likely to have trained on a substrate they would encounter in competition and might be more adaptable to changes in biomechanics which vary with substrate. However, there was no significant effect of substrate diversity on the incidence of training injury.

Training on a diverse set of substrates did appear to impact perception of performance. Respondents whose dogs trained on ≥4 substrates were significantly more likely to notice MDPs when their dogs trained on natural grass, sand, and dirt. Handlers with more experience observing their dog on a variety of substrates may be better at identifying MDPs, particularly subtle ones. For 8 of the 9 substrates, dogs training on ≥4 substrates were more often reported to have at least one MDP on a given substrate. For example, 51.5% (141/274) of dogs with experience training on ≤3 substrates were reported to have at least one MDP on natural grass, while 81.8% (27/33) of dogs with experience on ≥4 substrates were reported to have at least one MDP on natural grass. It is also possible that dogs training on more limited substrates have a performance advantage on those substrates, in comparison to dogs that train on a variety of substrates and need to adapt and recalibrate as the substrate changes. However, it is unknown whether this advantage, if real, is significant. A prospective study could be designed to evaluate the impact of substrate diversity on performance by challenging dogs to run the same obstacle course on substrates that they did and did not have experience training on, and having handlers evaluate for MDPs on each substrate.

Prior studies have utilized similar survey-based approaches to gather data from handlers, trainers, and owners of agility dogs (1–5, 11, 18, 28, 30, 38). Limitations of this study's retrospective, survey-based design include convenience sampling, selection bias, and response bias. Respondents were contacted in English, the survey was only available online, and most respondents were reached through existing association with the authors' social media accounts, or were already members of email lists and newsletters targeted toward canine agility professionals. The study population therefore does not represent a random selection of all canine agility handlers, owners, or trainers. Additionally, participants were allowed to respond to the survey multiple times to provide information about multiple dogs, but the majority (98.3% [292/300]) did not.

To minimize recall bias, we did not ask respondents to provide the exact hours in the training or competition schedule, or percentage time breakdown of substrate use at the time of the injury. The effect of level of competition and years in competition on the incidence of a particular CI could not therefore be assessed. Older dogs were more likely to have had a history of TI and CI, by virtue of having had more time to sustain these injuries. However, we did not evaluate the impact of the dog's age at the time the injury occurred, due to inability to separate out confounding variables that this survey did not assess, such as prior injuries or chronic conditions.

Similarly, respondents were asked to focus on one injury, in an attempt to reduce recall bias by allowing participants to select the injury with which they would be most likely to fully and accurately complete the survey. However, as previously discussed, this format introduces selection bias. Alternative strategies, however, also have their potential drawbacks—for example, asking participants to provide data regarding all prior injuries would significantly increase survey length, potentially decrease the number of overall responses or result in incomplete entries, and/or increase recall bias. Asking participants to focus on the dog's most recent injury would disregard the chronic impact of prior injuries as a potential confounding variable.

A perceived association between injury and substrate does not confirm that the injury was caused by the substrate; the injury may have been pre-existing and exacerbated by the substrate, or the injury may have been unrelated to the substrate. Respondents with strong pre-existing beliefs that substrates influence their dog's performance or cause injury may also have been more likely to complete to the survey, and to do so in reference to a particular injury perceived to be related to a substrate. Respondents with prior poor experiences on a certain substrate (such as observation of performance deficits in their dog, or performance deficits / injuries in another dog) could also be more likely to infer causation when an injury is observed to occur on that substrate.

This study provides evidence that a higher proportion of training injuries occur on rubber mat compared to other substrates, when controlling for the relative prevalence of substrates in use. Rubber mat was also perceived by respondents to be associated with injury and decreased performance. Dogs were perceived to perform best on natural grass and dirt. This study also demonstrates that the majority of agility dog owners, handlers, and trainers ground substrate to have an important impact on their dog's performance and potential for injury, and that owners' and handlers' decisions to train or compete at certain venues are influenced by their dogs' prior experiences on that substrate. The majority of respondents implemented changes to their dog's routine following injury, including changes in substrate use and modifications to training or competition regimen, rehabilitation, or other activities, sometimes lifelong.

Further research is needed to investigate the role of substrate composition and maintenance in canine agility injury and performance. Future studies can inform evidence-based recommendations for substrate use in training and competition, in order to maximize athletic performance and minimize the risk of injury in agility dogs.

## Data availability statement

The original datasets presented in this article are not made publicly available, as they contain identifying information of participants and agility dogs. A dataset with all owner and patient identifying information redacted (including owner name and contact information, and patient name, breed, sex, reproductive status, age, location, and list of trials entered) can be provided by the corresponding author upon request. Requests to access this dataset should be directed to isabeljimenezdvm@gmail.com.

## Ethics statement

Ethical review and approval was not required for the animal study because this study was entirely survey-based and utilized responses provided by owners and handlers of dogs. Written informed consent was obtained from the owners for the participation of their animals in this study.

## Author contributions

SC and MP distributed the survey. IJ collected the data and performed statistical analysis. IJ and SC wrote the manuscript. All authors provided critical feedback on the analysis and manuscript. All authors conceived the study idea and developed the internet-based survey.

## Funding

This study was performed at Veterinary Orthopedic and Sports Medicine Group in collaboration with Canapp Sports Medicine. Publication fees were funded by Canapp Sports Medicine.

## Conflict of interest

Author MP is employed by Clean Run Magazine, a canine agility publication. Author SC is the co-founder of Canapp Sports Medicine LLC, a virtual platform providing education on canine sports medicine. The remaining author declares that the research was conducted in the absence of any commercial or financial relationships that could be construed as a potential conflict of interest.

## Publisher's note

All claims expressed in this article are solely those of the authors and do not necessarily represent those of their affiliated organizations, or those of the publisher, the editors and the reviewers. Any product that may be evaluated in this article, or claim that may be made by its manufacturer, is not guaranteed or endorsed by the publisher.
