# Corrigendum: Internet-based survey evaluating the impact of ground substrate on injury and performance in canine agility athletes

**DOI:** 10.3389/fvets.2023.1130146

**Published:** 2023-03-31

**Authors:** Isabel A. Jimenez, Sherman O. Canapp, Monica L. Percival

**Affiliations:** ^1^Veterinary Orthopedic and Sports Medicine Group, Annapolis Junction, MD, United States; ^2^Department of Molecular and Comparative Pathobiology, The Johns Hopkins University School of Medicine, Baltimore, MD, United States; ^3^Canapp Sports Medicine LLC, Oakland, MD, United States; ^4^Clean Run Magazine, South Hadley, MA, United States

**Keywords:** canine agility, agility, medial shoulder syndrome, orthopedics, sports medicine, substrates, injury

In the published article, there was an error in Table 1 as published.

“Substrate diversity (<3 substrates vs. ≥4 substrates)” should have read “Substrate diversity (≤3 substrates vs. ≥4 substrates)”. The corrected Table 1 and its caption appear below.

**Table 1:** Relative risk for training injury and competition injury in canine agility athletes.

**Training injuries**		
**Parameter**	**RR** ^*^	***p*** **-value** ^†^
Age (< 6 years old vs. ≥6 years old)	1.2558	0.1809
Sex (female vs. male)	0.9611	0.8111
Concurrent sports (No vs. Yes)	0.7989	0.1491
Substrate diversity (≤ 3 substrates vs. ≥4 substrates)	1.0593	0.8465
**Competition injuries**		
**Parameter**	**RR** ^*^	***p*** **-value** ^†^
History of training injury (No vs. Yes)	2.2846	0.0005
Age (< 6 years old vs. ≥6 years old)	5.7935	< 0.00001
Sex (female vs. male)	0.9904	1.0000
Concurrent sports (No vs. Yes)	1.0267	1.0000
Substrate diversity (≤ 3 substrates vs. ≥4 substrates)	1.1795	0.6362

* Relative Risk (RR) was defined as the proportion of dogs with a history of injury that were positive for the parameter of interest, relative to the proportion of dogs with a history of injury that were negative for the parameter of interest. RR was calculated from 2 × 2 contingency tables.

† The significance level was α = 0.05. The p-value was calculated using the Fisher's Exact Test.

In the published article, there were two errors in the main text.

A correction has been made to the section Results, Substrate diversity and modifications to substrate use based on performance, paragraph 1.

This sentence previously stated:

“When substrate diversity was divided into binary categories—dogs with less substrate diversity (<3 substrates in training regimen) vs. dogs with more substrate diversity (≥4 substrates in training regimen), there was no impact of substrate diversity on the Relative Risk of TI or CI (Table 1).”

The corrected sentence appears below:

“When substrate diversity was divided into binary categories—dogs with less substrate diversity (≤ 3 substrates in training regimen) vs. dogs with more substrate diversity (≥4 substrates in training regimen), there was no impact of substrate diversity on the Relative Risk of TI or CI (Table 1).”

A correction has been made to the section Discussion, paragraph 15.

This sentence previously stated:

“For example, 51.5% (141/274) of dogs with experience training on <3 substrates were reported to have at least one MDP on natural grass, while 81.8% (27/33) of dogs with experience on ≥4 substrates were reported to have at least one MDP on natural grass.”

The corrected sentence appears below:

“For example, 51.5% (141/274) of dogs with experience training on ≤3 substrates were reported to have at least one MDP on natural grass, while 81.8% (27/33) of dogs with experience on ≥4 substrates were reported to have at least one MDP on natural grass.”

The authors apologize for these errors and state that this does not change the scientific conclusions of the article in any way. The original article has been updated.

