# Supplementary material for: Internet-based survey evaluating the impact of ground substrate on injury and performance in canine agility athletes
Source: Front Vet Sci. 2022 Oct 17;9:1025331. doi: 10.3389/fvets.2022.1025331 (PMC9624126; doi:10.3389/fvets.2022.1025331)
Supplement: Supplementary file 1 [file Data_Sheet_1.PDF]

Supplementary Material

1 Supplementary Tables

Supplementary Table S1: Substrate distribution in the training regimen of canine agility athletes (n=307).

Substrate	# of dogs	% of dogs	% time on substrate*
Natural grass	262	85.3%	51.8%
Artificial turf	156	50.8%	50.9%
Dirt	106	34.5%	32.1%
Sand	66	21.5%	24.8%
Rubber mat	50	16.3%	33.0%
Foam mat	41	13.4%	36.1%
Wood mulch	11	3.6%	39.8%
Poured rubber	1	0.33%	10.0%
Other	24	7.8%	
Engineered wood fiber	1	0.33%	50.0%
Stone / gravel	6	1.95%	42.5%
Sand mixtures	13	3.91%	31.5%
Dirt mixture	1	0.33%	90.0%
Clay	1	0.33%	4.0%
Silica	1	0.33%	1.0%
Lime with rubber infill	1	0.33%	5.0%

* Percent of time on substrate was calculated as the average proportion of time that owners reported their dog spent training on that substrate within the training regimen, among dogs who reported training on the substrate. Data from 295 dogs was included; of respondents who provided data that did not add up to 100%, 12 did not respond to a request to correct their data and these results were excluded.

Supplementary Table S2: Incidence Proportion of each substrate and perceived influence of substrate on training injury (n=109).

					Perceived relationship between substrate and TI					
					Definitely related		Unsure if related		Not related	
**Substrate**	**Dogs injured on substrate (n_i)**	**% of total TIs**	**Dogs training on substrate (n_t)**	**IP***	**#**	**%**	**#**	**%**	**#**	**%**
Do not remember	11	10.09%	-	-	0	0.00%	4	36.36%	7	63.64%
Rubber mat	16	14.68%	50	32.00%	14	87.50%	1	6.25%	1	6.25%
Artificial turf	24	22.02%	156	15.38%	7	29.17%	6	25.00%	11	45.83%
Sand	10	9.17%	66	15.15%	5	50.00%	1	10.00%	4	40.00%
Natural grass	35	32.11%	262	13.36%	8	22.86%	8	22.86%	19	54.29%
Wood mulch	1	0.92%	11	9.09%	0	0.00%	0	0.00%	1	100.00 %
Foam mat	3	2.75%	41	7.32%	0	0.00%	1	33.33%	2	66.67%
Dirt	4	3.67%	106	3.77%	1	25.00%	1	25.00%	2	50.00%
Other	5	4.59%	-	-	3	60.00%	1	20.00%	1	20.00%

* For each substrate, Incidence Proportion (IP) was defined as the proportion of dogs sustaining TI (n_i) out of the total number of dogs reported to train on that substrate (n_t) and is intended to account for differences in the overall number of dogs training on each substrate.

Supplementary Table S3: Perceived influence of obstacle on training injury (n=109).

Obstacle	# of dogs with associated TI	% of total TIs (n=109)	% of obstacle-related TIs (n=64)
Jumps	24	22.02%	37.50%
Contacts	19	17.43%	29.70%
A Frame	7	6.42%	10.94%
Dog Walk	7	6.42%	10.94%
Teeter	3	2.75%	4.69%
Pause Table	0	0%	0%
Weaves	13	11.93%	20.31%
Tunnel	7	6.42%	10.94%
Backside	1	0.92%	1.56%

Supplementary Table S4: Perceived influence of substrate on competition injury (n=59).

Substrate	Dogs injured on substrate	% of total CIs	Perceived relationship between substrate and CI					
			Definitely related		Unsure if related		Not related	
			#	%	#	%	#	%
Do not remember	2	3.39%	0	0.00%	1	50.00%	7	50.00%
Dirt	22	37.29%	9	40.91%	4	18.18%	9	40.91%
Natural grass	15	25.42%	4	26.67%	3	20.00%	8	53.33%
Artificial turf	14	23.73%	6	42.86%	3	21.43%	5	35.71%
Rubber mat	4	6.78%	3	75.00%	1	25.00%	0	0.00%
Sand	2	3.39%	2	100.00%	0	0%	0	0%
Foam mat	0	0%	-	-	-	-	-	-
Wood mulch	0	0%	-	-	-	-	-	-
Pea gravel	0	0%	-	-	-	-	-	-
Poured rubber	0	0%	-	-	-	-	-	-

Supplementary Table S5: Perceived influence of obstacle on competition injury (n=59).

**Obstacle**	**Dogs reporting CI associated with obstacle**	**% of total CI (n=59)**	**% of obstacle-related CI (n=36)**
Jumps	10	16.95%	27.78%
Tunnel	9	15.25%	25.00%
Contacts	8	13.56%	22.22%
A Frame	5	8.47%	13.89%
Dog Walk	1	1.69%	2.78%
Teeter	1	1.69%	2.78%
Pause Table	1	1.69%	2.78%
Weaves	5	8.47%	13.89%
Other	4	6.78%	11.11%
Turn	1	1.69%	2.78%
Tire	1	1.69%	2.78%
Wall	1	1.69%	2.78%
Chute	1	1.69%	2.78%

2 Supplementary Figures

	Substrates									
MDP	Grass	Turf	Sand	Dirt	Foam mat	Rubber mat	Wood mulch	Pea gravel	Poured rubber	***Total***
Poor grip / slipping prior to jump takeoff	0	1	0			1				2
Taking off closer to the obstacle than usual	0	0	0			0				0
Shoulder-plant or face-plant when landing a jump	0	0	0			1				1
Slipping when entering or exiting tunnels at speed	0	1	0			1				2
Pulling out of turns	0	0	0			0				0
Trouble with footwork in the weave poles	0	0	0			1				1
Difficulty making weave pole entries	0	0	0			1				1
Knocking more bars than usual	0	0	0			0				0
Slower time than usual	0	0	0			0				0
Shortened stride length	0	1	0			1				2
Increased soreness after training or competition	0	0	0			0				0
Total	0	3	0			6				***9***

Supplementary Figure 1. Sample performance matrix, evaluating MDPs noted during agility training or competition on different substrates. The owner indicated in their survey that the dog's current training regimen includes natural grass, sand, and rubber mat, and specified that MDPs has been noted on artificial turf and rubber mat. No MDPs had been observed by the owner on grass. Substrates on which the dog’s performance has not been evaluated were excluded and grayed out in the table.

<u>Survey: Finding The Right Footing</u>

Investigating the impact of ground material on performance and injury in agility dogs

1. **Owner Contact Information**
    - Name
    - Role (owner, handler, trainer)
    - City / Town (optional)
    - State / Province
    - Country
    - Email address
    - Phone number (optional)

2. **Dog Information**
    - Registered name
    - Breed
    - Male/Female
    - Intact/Neutered
    - Approximate age (include years + months)

Please answer the following questions regarding your dog's training in a typical year.

3. **Training Regimen:**
    - ☐ How many months out of the year does your dog train on agility obstacles? (Example: 3 months per year)
    - ☐ How many days per week does your dog train on agility obstacles?
    - ☐ How many hours per day does your dog train on agility obstacles?
    - ☐ How many full courses do your run in training each week?

4. **What <u>percentage</u> of training time does your dog spend <u>training indoors</u> (compared to outdoors)?**

5. **Please list the <u>percentage</u> of time that your dog spends <u>TRAINING</u> on each of the following surfaces. For surfaces that your dog does NOT train on, please enter 0%. The total should add up to 100%.**

**Surface**	**Percentage**
Natural grass	
Artificial turf	
Sand	
Dirt	
Foam matting	
Rubber matting	
Wood mulch	
Pea gravel	
Poured rubber	
Other (please describe)	

6. **What region does your dog TRAIN in?**
   - ☐ North America
   - ☐ Europe
   - ☐ Australia
   - ☐ South America
   - ☐ Africa
   - ☐ Asia

7. **Has your dog ever sustained an injury while training? If your dog has sustained multiple injuries, please choose one to focus on for the following questions.**
   - ☐ Yes
   - ☐ No

8. **Injuries sustained during <u>TRAINING</u> *(skip this question if your dog has not sustained an injury)***
    a. What was the official diagnosis, if any?
    b. What substrate was your dog training on when the injury was sustained?
    c. Do you feel that the substrate contributed to the injury? If so, please explain.
    d. Was the injury associated with a particular obstacle in the course? If so, please explain.

A reference image was provided to respondents showing a dog with labeled body sites.

9. **For the injury described in question 8, which body site was affected?**

☐ Neck	☐ Hip
☐ Shoulder	☐ Stifle
☐ Elbow	☐ Tarsus (hock)
☐ Carpus	☐ Iliopsoas (groin) muscle
☐ Forelimb digits	☐ Hindlimb digits
☐ Lower back	☐ Other (please specify)

10. **For the injury described in question 8, if the injury was sustained to a limb, which limb was affected?**
    - ☐ Left front limb
    - ☐ Right front limb
    - ☐ Left hind limb
    - ☐ Right hind limb
    - ☐ Not applicable (injury did not affect the limbs)

Please answer the following questions about your dog in competition in a typical year:

11. **Competition information:**
    - ☐ How many years has your dog been competing in agility?
    - ☐ How many years has your dog been competing at their current level?
    - ☐ How many trials per year does your dog typically enter?
    -

12. **What region does your dog COMPETE in?**
    - ☐ North America
    - ☐ Europe
    - ☐ Australia
    - ☐ South America
    - ☐ Africa
    - ☐ Asia

13. What agility level does your dog compete at?

- ☐ Beginners/Intro
- ☐ Starters/Novice
- ☐ Open/Advanced
- ☐ Excellent
- ☐ Masters/Senior
- ☐ Champion
- ☐ Other (please specify)

14. What agility Classes does your dog compete at?

- ☐ Standard Agility
- ☐ Jumping
- ☐ Games / Nonstandard Classes
- ☐ Tournaments
- ☐ Premier / International / Masters Challenge
- ☐ Other (please specify)

15. What jump height division does your dog compete in (for example, 12 in Select or 22 inch Championship)?

16. Does your dog concurrently participate in any other canine sports? If so, which sports? Please be specific (field, obedience, show, etc.)

17. What trials does your dog participate in? Choose all that apply.

- ☐ AAC (Agility Association of Canada)
- ☐ ADAA (Agility Dog Association of Australia)
- ☐ AKC (American Kennel Club)
- ☐ ASCA (Australian Shepherd Club of America)
- ☐ BAA (British Agility Association)
- ☐ CKC (Canadian Kennel Club)
- ☐ FCI (Federation Cynologique Internationale)
- ☐ CPE (Canine Performance Events)
- ☐ KC (UK Kennel Club)
- ☐ NADAC (North American Dog Agility Council)
- ☐ NZKC (New Zealand Kennel Club)
- ☐ TDAA (Teacup Dogs Agility Association)
- ☐ UKA (United Kingdom Agility)
- ☐ UKC (United Kennel Club)
- ☐ UKI (UK Agility International)
- ☐ USDAA (United States Dog Agility Association)
- ☐ Other (please specify)

18. Has your dog ever sustained an injury during competition? If your dog has sustained multiple injuries, please choose one to focus on for the following questions.
- ☐ Yes
- ☐ No

19. Injuries sustained during COMPETITION *(skip this question if your dog has not sustained an injury)*:
   a. What was the official diagnosis, if any?
   b. What substrate was your dog competing on when the injury was sustained?
   c. Do you feel that the substrate contributed to the injury? If so, please explain.
   d. Was the injury associated with a particular obstacle in the course? If so, please explain.
   e. What agility level was your dog competing at when the injury was sustained?
   f. What agility Class was your dog competing in when the injury was sustained?

20. For the injury described in question 18, which body site was affected?
- ☐ Neck
- ☐ Shoulder
- ☐ Elbow
- ☐ Carpus
- ☐ Forelimb digits
- ☐ Lower back
- ☐ Hip
- ☐ Stifle
- ☐ Tarsus (hock)
- ☐ Iliopsoas (groin) muscle
- ☐ Hindlimb digits
- ☐ Other (please specify)

21. For the injury described in question 18, if the injury was sustained to a limb, which limb was affected?
- ☐ Left front limb
- ☐ Right front limb
- ☐ Left hind limb
- ☐ Right hind limb
- ☐ Not applicable (injury did not affect the limbs)

22. Has your dog's training or competition regimen changed since the injuries described above were sustained? Please include information regarding changes in training or competition regimen.

23. **Please rank the following substrates based on your dog's performance on that substrate, with #1 being the surface on which your dog performs BEST. *For any surfaces that do not apply to your dog, select N/A.***
    - Natural grass
    - Artificial turf
    - Sand
    - Dirt
    - Foam Matting
    - Rubber Matting
    - Wood Mulch
    - Pea gravel
    - Poured rubber
    - Other (please describe below)

24. **If your dog performs best on a substrate other than the ones listed in question 23, please describe the substrate here.**

25. **Has your decision to train on the above substrates changed over time or been influenced by injuries sustained during training or competition? Please explain.**

26. For surfaces that your dog currently or has previously trained / competed on, please check the appropriate box if you have noticed any of the following indicators of DECREASED athletic performance while your dog was training or competing on that surface. ***If your dog has never trained on the substrate, check N/A indicating that performance has never been assessed on that substrate.***

	Natural grass	Artificial turf	Sand	Dirt	Foam mat	Rubber mat	Wood mulch	Pea gravel	Poured rubber
Poor grip/slipping prior to jump takeoff									
Taking off closer to the obstacle than usual									
Shoulder-plant or face plant when landing a jump									
Slipping when entering or exiting tunnels at speed									
Pulling out of turns									
Trouble with footwork in the weave poles									
Difficulty making weave pole entries									
Knocking more bars than usual									
Slower time than usual									
Shortened stride length									
Increased soreness after training or competition									
N/A – my dog has never trained on this surface									

27. Please include any additional comments (optional).
