# Supplementary figures and images for: Internet-based survey evaluating the impact of ground substrate on injury and performance in canine agility athletes
Source: Front Vet Sci. 2022 Oct 17;9:1025331. doi: 10.3389/fvets.2022.1025331 (PMC9624126; doi:10.3389/fvets.2022.1025331)

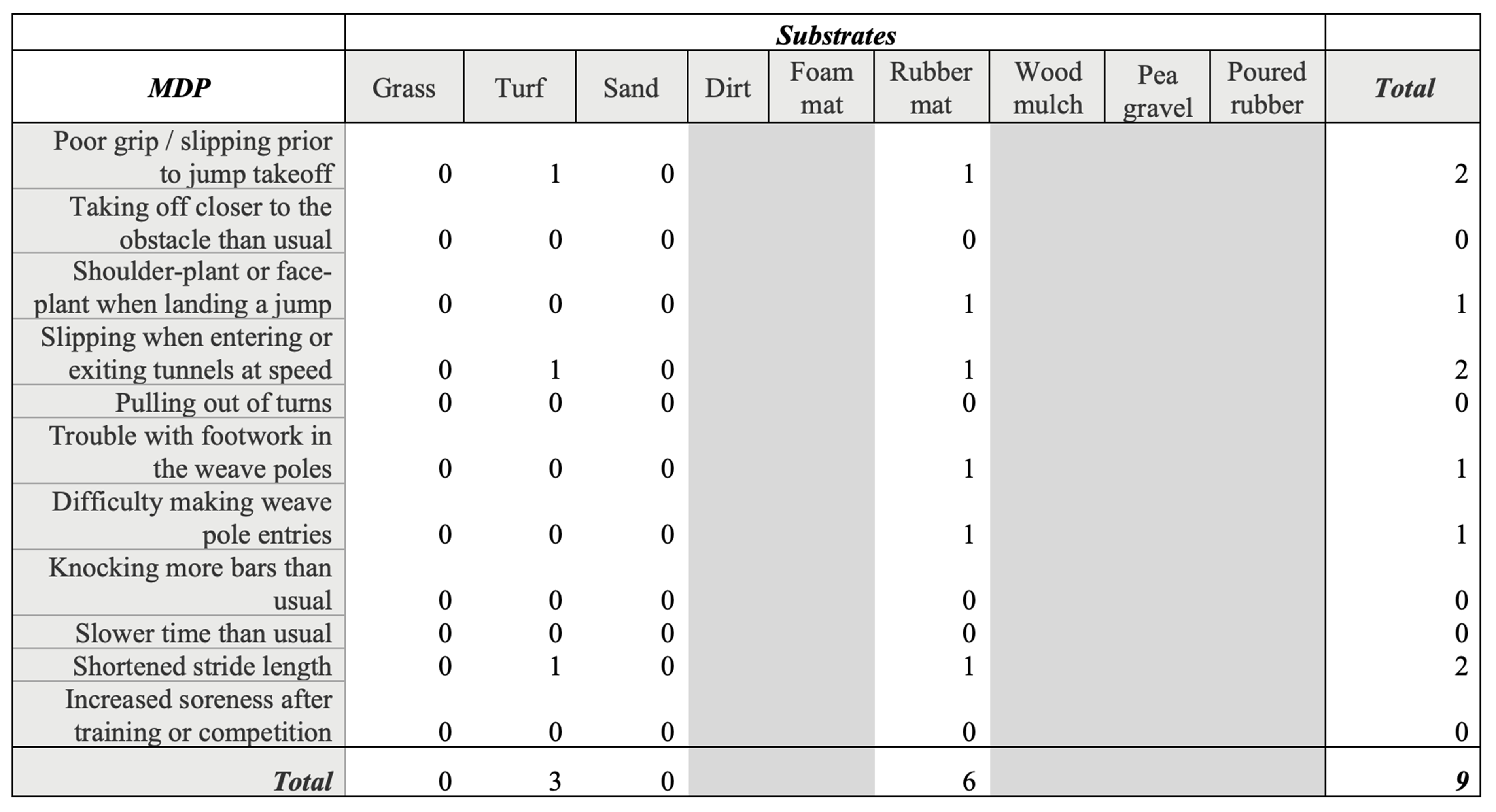

Supplement: Supplementary file 3 [file Image_1.PNG]
